# MYC function and regulation in physiological perspective

**DOI:** 10.3389/fcell.2023.1268275

**Published:** 2023-10-24

**Authors:** Rajiv Kumar Jha, Fedor Kouzine, David Levens

**Affiliations:** Gene Regulation Section, Laboratory of Pathology, Center for Cancer Research, National Cancer Institute (NCI), Bethesda, MD, United States

**Keywords:** MYC, transcription, transcription-amplifier, MYC function, MYC regulation, MYC-inhibitors, DNA-topology

## Abstract

MYC, a key member of the Myc-proto-oncogene family, is a universal transcription amplifier that regulates almost every physiological process in a cell including cell cycle, proliferation, metabolism, differentiation, and apoptosis. MYC interacts with several cofactors, chromatin modifiers, and regulators to direct gene expression. MYC levels are tightly regulated, and deregulation of MYC has been associated with numerous diseases including cancer. Understanding the comprehensive biology of MYC under physiological conditions is an utmost necessity to demark biological functions of MYC from its pathological functions. Here we review the recent advances in biological mechanisms, functions, and regulation of MYC. We also emphasize the role of MYC as a global transcription amplifier.

## Introduction

The Myc gene was first identified in the early 1980s as a cellular homolog of the retroviral v-Myc oncogene (Duesberg et al., 1977; Sheiness et al., 1978; Conacci-Sorrell et al., 2014). Its discovery led to intense research efforts to understand its function and deregulation in cancer. MYC deregulation was soon associated with genomic rearrangements including translocations in Burkitt lymphoma, gene amplification and chromosomal circles in leukemia and carcinoma, and deregulation by HPV insertion in cervical carcinoma (Dalla-Favera et al., 1982; Taub et al., 1982; Spencer and Groudine, 1991; Wasylishen and Penn, 2010; Adey et al., 2013). Subsequently, mutations that stabilize MYC protein and mRNA were recognized in malignancy (Dang, 2012). Because all these situations occur in an oncogenic setting, thousands of studies explored the cellular consequences of MYC overexpression. Upon discovering that the basic-helix-loop-helix (bHLH) protein MYC dimerizes with its bHLH partner Myc-associated factor-X referred to as MAX and binds with E-boxes (5′-CACGTG-3′) and presumed to activate transcription, the principal focus of studies to define the pathologic role of MYC revolved upon the identification of its transcriptional targets (Blackwood and Eisenman, 1991; Grandori and Eisenman, 1997; Eilers and Eisenman, 2008; Dang, 2012). The notion was that MYC programmed the expression of a discrete set of mRNAs that bypassed normal growth control leading to unrestrained proliferation. Most of these studies exploited a variety of transformed and tumor cell lines to explore pathologic MYC function. Fewer studies focused on physiological role of MYC. In the untransformed, non-oncogenic situation, MYC was found to be an immediate early gene, turned off during the G0-stationary phase of the cell cycle, but upregulated transiently during the transition to G1/S (Kelly et al., 1983; Armelin et al., 1984; Wang et al., 2008). Upon entering steady-state cell-cycle growth, MYC was stably expressed at lower levels until growth once again arrested. Survey of mRNA expression indicated that while MYC upregulated the expression of many genes involved in cell cycle progression, it also repressed a small number of cell-cycle antagonists (Bretones et al., 2015). Sustained high level expression of MYC elicited apoptosis in non-transformed cells and so could not be maintained (Evan et al., 1992; Murphy et al., 2008). In the bulk of this review, we will consider the biological mechanisms and functions of MYC in non-transformed cells, tissues, and organisms. A description of this physiology is essential to distinguish whether the oncogenic actions of MYC arise due to an exaggeration of its normal functions or whether high level expression conjures new modes and mechanisms of MYC activity otherwise unseen.

## MYC domain organization and function

The MYC family of proteins consists of three paralogs, MYC (c-MYC), MYCN (N-MYC) and MYCL (L-MYC) (Brodeur et al., 1984; Kohl et al., 1984; Nau et al., 1985). Although MYC family genes encode proteins with similar structural architecture and function, each MYC paralog is located on a different chromosome (MYCL, MYCN and MYC are in chromosomes 1, 2, and 8 respectively) and expressed at distinct times and locations during cellular differentiation (Dalla-Favera et al., 1982; Schwab et al., 1984; Zelinski et al., 1988; Ruiz-Pérez et al., 2017; Llombart and Mansour, 2022). MYCN and MYCL have tissue-specific function. MYCL is expressed and functions in dendritic cells, gastrointestinal cells, and lung cells. MYCN is expressed in neural and neuroendocrine tissue and is critical for the development of nervous system (Llombart and Mansour, 2022). MYC is composed of 439 amino acids and contains an N-terminal transactivation domain (TAD), and a C-terminal DNA-binding domain. The TAD (residue 1-143) forms an intrinsically disordered domain and is necessary for biological activity of MYC and MYC-mediated transcriptional activation (Kato et al., 1990). The C-terminal domain comprises ∼80 residues and consists of a bHLH -leucine zipper (bHLH-ZIP) segment from residues 357-439. The bHLH-ZIP domain forms specific heterodimers with MAX (Blackwood and Eisenman, 1991; Amati et al., 1992; Amati et al., 1993; Kato et al., 1992). This interaction facilitates the ability of MYC’ to bind DNA with preference, but not absolute specificity, for binding to the canonical E-box (5′-CACGTG-3′) (Blackwood and Eisenman, 1991; Guo et al., 2014; Carroll et al., 2018). Besides sequence recognition, a major component of MYC recruitment to the DNA are its interactions with the transcription machinery at accessible promoters (Guo et al., 2014). Initially MYC seemed to bind a wide range (2,500–25,000) of sites throughout the genome that varied according to cell type (Cawley et al., 2004). Classification and functional assessment of the programs regulated by MYC between different tissues and cells seemed complex and somewhat incoherent. The number of MYC peaks was significantly affected by the arbitrary threshold chosen to distinguish real peaks from the background and experimental conditions that most often lacked an internal control, such as “spike” chromatin from a heterologous genome, to improve quantitation (Bonhoure et al., 2014). Moreover, the normalization of gene mRNA output obscured the observation of global transcription amplification by MYC, with sensitivity to the artificial threshold used to differentiate “real” from non-specific binding (Lovén et al., 2012).

Upon binding at promoters, the transregulatory domains of MYC and its isoforms, are believed to project its influence onto target genes through patches of amino acids that share high sequence homology among the three MYC isoforms. These patches are referred to as MYC boxes (MBs). From the amino-to carboxyl terminus, there are six conserved MBs: MB0, I, II, IIIa, IIIb, and IV. They are generally unstructured and can adopt specific conformations induced upon partner-protein binding. The degree of plasticity for each MB upon complexing with different partners has not been explored. The inventory and functional roles for MB-interacting partners that have been most intensively investigated are involved in transcription and chromatin process, or control MYC turnover, has recently been reviewed (Das et al., 2023).

A sampling of the MYC-interactome shows MB0 interactions with general transcription factor TFIIF (Kalkat et al., 2018). MBI and MBII reside within the TAD and are critical for transcriptional and cell-transforming functions of MYC. MYC box I controls its proteasome mediated degradation of MYC proteins (Farrell and Sears, 2014). Aurora A, independent from its kinase activity, interacts with MBI to stabilize MYC (Dauch et al., 2016). MBII plays a crucial role in recruiting MYC transactivation coactivators such as TRRAP, GCN5, TIP48, TIP49, TIP60, CBP/p300, and SKP2 (Adhikary and Eilers, 2005; Conacci-Sorrell et al., 2014; Tu et al., 2015; García-Gutiérrez et al., 2019). Because TRRAP is a protein that participates in multiple large protein complexes engaged in chromatin remodelling and histone acetylation (Zhang et al., 2014), it may impart multiple functions when joined with a promoter-bound MYC. The central region of MYC containing MBIII and MBIV starts with a proline-rich PEST segment, followed by a calpain cleavage site (CAPN); the N-terminal fragment of this cleavage, known as “MYC-nick,” lacking the nuclear localization signal (NLS) situated to the C-terminal side of the cleavage site, resides in the cytoplasm and participates in interactions and functions of the cytoskeleton (Conacci-Sorrell et al., 2010; Anderson et al., 2016). MBIII is important for transcriptional repression (Kurland and Tansey, 2008; Garcia-Sanz et al., 2014), but also interacts with WDR5 (a scaffolding protein that nucleates the assembly of histone modifier complex) and facilitates histone H3 Lys4 (H3K4) methylation which is thought to increase the interaction of MYC with active promoters (Thomas et al., 2015). MBIV is necessary for transcriptional activity of MYC and induction of apoptosis (Cowling et al., 2006) and has been shown to interact with the transcriptional coregulator HCF-1 (Thomas et al., 2016). Although each of the MBs interact with multiple partners and have been shown to modulate MYC activity, the precise role of individual MBs has not been fully ascribed.

## MYC, an amplifier of transcription

Transcription activation involves the binding of transcription factors to specific DNA sequences, which recruit the transcriptional machinery, coactivators, and chromatin modifiers to form a transcriptional complex that initiates gene transcription. Transcription factors can recruit coactivators such as CBP/p300, which possess histone acetyltransferase activity and can acetylate histones to promote an open chromatin structure that allows for gene transcription. In addition, transcription factors can recruit chromatin modifiers such as SWI/SNF, which can remodel chromatin to allow access to the transcriptional machinery (Bannister and Kouzarides, 2011). Unlike transcription activation, transcription amplification refers to the process by which transcription factors globally enhance the expression of all active genes in the cell (Lin et al., 2012; Nie et al., 2012; Li et al., 2013). Transcription amplification is different from gene amplification where the number of copies of a specific gene increases without an increase in the transcription output of each copy. Gene amplification can result from DNA replication errors, chromosome translocations or gene rearrangements (Albertson, 2006; Beroukhim et al., 2010; Matsui et al., 2013; Schaub et al., 2018). In contrast, transcription amplification occurs through the recruitment of coactivator complexes or other factors that enhance the efficiency of transcriptional reinitiation and elongation, and so increase the number of RNA polymerases (RNAP) that are engaged in transcription (Wolf et al., 2015). Transcription amplification enhances the expression of a gene beyond what would be expected based on the level of transcription factor binding alone. While it was initially believed that MYC acted as a sequence-specific transcription factor, turning on genes via E-boxes (Blackwell et al., 1990; Halazonetis and Kandil, 1991; Kerkhoff et al., 1991; Prendergast and Ziff, 1991), an alternate model has been posed in which MYC acts as a global amplifier of all active genes (Lin et al., 2012; Lovén et al., 2012; Nie et al., 2012; Nie et al., 2020; Wolf et al., 2015).

**FIGURE 1:**
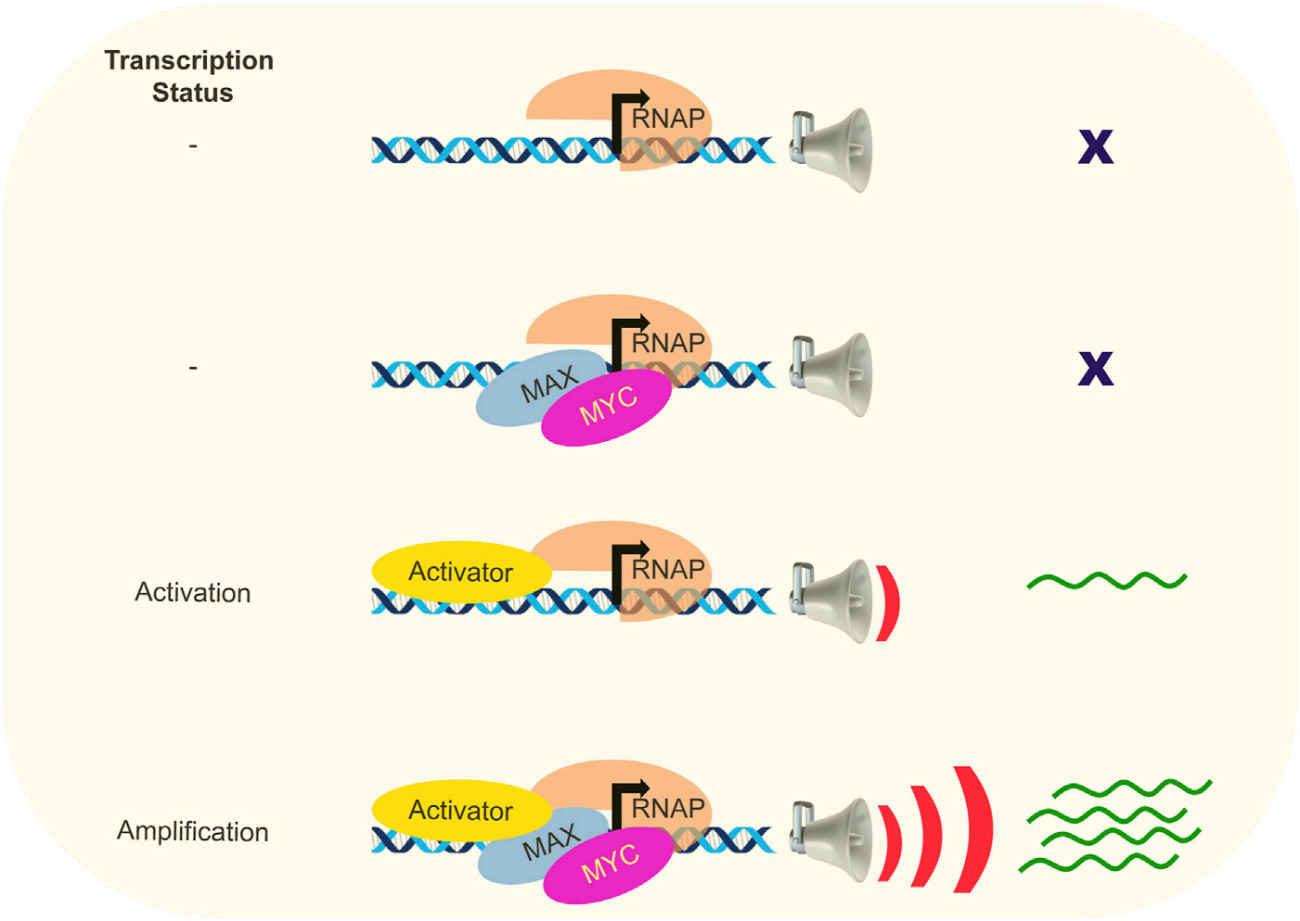
MYC is an amplifier of transcription. Schematic representation of the role of MYC as transcription amplifier is depicted. MYC exerts its influence on actively transcribed genes in the presence of activators, rather than being involved in transcriptional processes at silent genes. When MYC is not involved, activator can start transcription albeit with low outputs. Participation of MYC leads to an augmentation of gene expression beyond what would be typically anticipated solely based on the binding of transcription factors.

When viewed a transcriptional activator, the expectation and goal were to identify specific, direct MYC target genes to provide insights into the crucial downstream targets and biological processes responsible for mediating the physiological functions and oncogenic pathology of MYC. Numerous studies were undertaken to identify MYC-regulated genes by employing techniques such as microarray or next-generation sequencing to compare RNA expression profiles and genome-wide mapping of MYC-bound chromatin. The notion that MYC and MYC-MAX complexes regulate a limited and well-defined set of target genes for their various roles has been largely challenged (Orian et al., 2003; Ji et al., 2011; Lee et al., 2012; Hurlin, 2013; Sullivan et al., 2022). Studies aimed to establish a universal signature of MYC target genes across cell types have been unsuccessful (Lee et al., 2012; Sullivan et al., 2022). Investigations across various cell types consistently revealed the presence of MYC proteins at nearly all promoters located in open chromatin regions (Chen et al., 2008). Moreover, a strong correlation between MYC binding and the presence of histone marks associated with open chromatin, particularly H3K4Me3 and H3K27Ac was observed (Nie et al., 2012). Conversely, MYC was excluded in the regions exhibiting repressive histone modifications. These results argued against the role of MYC as selective target (E box-dependent) transcription activator and led to further consideration of the transcription amplifier model, where MYC acts to globally enhance the expression of transcriptionally active genes in a nonlinear manner (Figure 1) (Lin et al., 2012; Nie et al., 2012). The transcriptional response of an active gene rises until output at the affected promoter saturates. This amplification is more efficient on highly transcribed genes, effectively raising their expression ceilings. MYC exhibited widespread binding to all promoters associated with RNAP II activity, resulting in a significant enhancement of transcription for a diverse repertoire of genes. MYC action does not entail the activation of novel genes; instead, it amplifies the expression levels of transcribed genes and so accelerates and amplifies ongoing cellular programs. Highly expressed MYC target genes tend to harbor canonical E boxes, but this is not obligatory and there is no strict correlation between MYC binding and the presence of E boxes for MYC- amplified genes in non-transformed cells (Nie et al., 2012).

**FIGURE 2:**
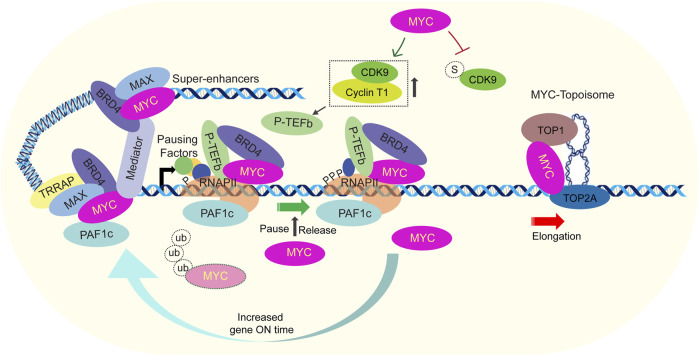
Current model for factors involved in transcription amplification by MYC. MYC interacts with essential transcription regulators involved in critical events at promoters, either coincidently or through regulated processes. MYC recruits key activators such as general transcription factors, Mediator, PAF1c, P-TEFb, DSIF, and exosome (other components omitted for simplicity). Once transcription starts, pausing factors interact with RNAP II near the start site, causing it to pause around 50 bp downstream from the initiation site. Together with cofactors like BRD4, MYC recruits P-TEFb which phosphorylates the pausing factors and RNAP II. MYC suppresses CDK9 sumolyation, facilitating active P-TEFb formation. MYC also recruits PAF1c and in association with HUWEI1-mediated ubiquitylation of MYC, PAF1c is transferred to RNAP II. These events collectively trigger the release of the paused transcription complex and initiate transcription elongation. Moreover, torsional stress generated due to transcription elongation is resolved by the MYC-Topoisome complex. It activates the catalytic activity of both TOP1 and TOP2A, helping to maintain DNA supercoiling homeostasis. MYC also extends the duration of residence times of transcription machinery like TBP, SPT5 and RNAP II and this leads to the extension of transcriptional bursts (gene ON time). These events help to explain the role of MYC as an amplifier of transcription.

The complexity of transcription amplification can be influenced biologically by input signals, cis-elements, other transcription factors, and analytically by the algorithms and pipelines used for analysis. These factors can highlight or obscure the relationship between MYC binding and promoter output in omics studies. To exclude such interfering or biased factors, minimal promoter and the reporter-based assay was designed to interrogate MYC function (Nie et al., 2020). Basal reporter expression was insensitive to MYC, and an initial activator signal was required to sensitize the promoter to MYC amplification to achieve increased transcriptional output. MYC boosted reporter gene expression to much higher levels than was attainable solely with saturating levels of transactivators. Further, MYC-mediated transcription amplification was severely attenuated by mutations in MBI and MBII but augmented by mutations in MBIII. This suggests that the MB regions coordinate with various proteins to control the chromatin opening and progression through the transcription cycle to achieve transcription amplification. The amplifier model for MYC functions is supported by the observation that MYC promotes transcription elongation by recruiting P-TEFb, PAF1c and super-elongation complexes (Jaenicke et al., 2016; Chen et al., 2017; Endres et al., 2021; Aoi et al., 2022). Increased MYC occupancy consequently led to increased P-TEFb with elevated levels of Serine 2 phosphorylation at RNAP II (a modification linked to elongation), escalated levels of elongating RNAP II, and augmented mRNA levels for active genes. Therefore, the main consequence of increased MYC is the amplification of transcription (Figure 2) (Rahl et al., 2010).

Although, it has been suggested that the binding of transcription factors to enhancer elements, super-enhancers, or other regions that drive the recruitment and activity of the transcription machinery plays a critical role in the non-linear mode of transcriptional amplification (Hnisz et al., 2016), the direct mechanism/s how MYC increases the output of expressed genes demands further investigation. A new report argues that a DNA-binding independent function of MYC helps it to function as a global amplifier (Guan et al., 2023). These authors report that MYC regulates P-TEFb availability through the inhibition of CDK9 sumoylation. CDK9 interacts with UBC9 and the PIAS family E3 ligase, specifically PIAS1, to promote CDK9 sumoylation. This modification impedes the binding between CDK9 and Cyclin T1, leading to the disruption of active P-TEFb assembly. MYC, through its independent interaction with CDK9 and UBC9/PIAS1, inhibits the association between CDK9 and UBC9/PIAS1, thereby preventing CDK9 sumoylation (Figure 2). By facilitating the formation of the P-TEFb complex, MYC enhances the phosphorylation of Ser2 on the RNAP II CTD, promoting global transcription amplification through transcriptional elongation (Figure 2). The full extent of transcriptional functions of MYC depends on both its local and global effects, as well as its interactions with various transcriptional cofactors. In addition, the differences in transcriptional profiling and transformation potency observed between full-length MYC and truncated MYCs (Yu et al., 2018; Guan et al., 2023). MYC also indirectly amplifies transcription by inducing the expression of GCN5 that acetylates histones (chromatin opening) and PRPS2 that promotes nucleotide biosynthesis (McMahon et al., 1998; McMahon et al., 2000; Knoepfler et al., 2006; Cunningham et al., 2014).

A recent report (Patange et al., 2022) investigated the transcriptional kinetics and mechanisms through which MYC enhances gene expression in living cells. A light-controlled MYC protein was translocated from the cytoplasm to the nucleus upon blue light illumination, thereby controlling MYC activity in human cells. Photo-activation and RNA imaging enabled precise measurements of gene regulation and the MYC action on transcription factor dynamics and transcription amplification. Single-molecule fluorescence *in situ* hybridization (smFISH) in fixed cells and MS2-tagging of RNA in live cells were used to assess the immediate impact of MYC on transcription bursting. The findings demonstrate that MYC influences the length of time that other core transcriptional factors reside at promoters. Elevated MYC levels uniquely influence the dwell time of various transcriptional machinery complexes. The glucocorticoid receptor (GR) remained unchanged, while SPT5, TBP, and RNAP II exhibited increased dwell time, and MED1, a mediator component, showed decreased dwell time. Elevated MYC enhanced RNA output from its target genes and alterations in burst duration were attributed to changes in the residency of transcriptional machinery and hence altered transcription output. Overall, MYC universally extended the duration of transcriptional bursts (increased gene ON time, i.e., transcriptionally active state), without altering their frequency (Figure 2) (Patange et al., 2022). Although bursting duration was preferentially enhanced for genes with lower expression, it should be noted that the highly expressed genes, most likely, were pre-saturated with endogenous MYC.

MYC is primarily associated with transcription amplification, however, many reports have revealed that it also represses several genes. Most repression may represent an algorithmic artefact of RNA normalization by programs such as DE-seq2 when comparing samples. Yet a small number of MYC repressed targets survive the normalization correction and are truly repressed. The precise mechanism underlying transcriptional repression of MYC is not fully understood. However, it seems that MYC uses surrogates to affect repression. For example, MYC exploits transcription factors like MIZ-1 (Myc-interacting zinc-finger protein 1) or SP1 that recruit corepressor, or changes in chromatin accessibility driven by epigenetic modifications which lead to the displacement of DNA-bound coactivators to ultimately achieve gene repression (Seoane et al., 2001; Kurland and Tansey, 2008; Wiese et al., 2013; Walz et al., 2014; Lourenco et al., 2021). Further, interaction of MYC with PAF1c forms a repressive complex, inhibiting function of PAF1c as an elongation factor (Jaenicke et al., 2016). It is important to rule out the potential involvement of indirect mechanisms of repression that involve ability of MYC to amplify the expression of negative regulators of transcription, such as repressor genes and other repressive components such as microRNAs (Wolf et al., 2015; Poole and van Riggelen, 2017). Consequently, the activation of these repressive components could ultimately result in the repression of target genes. For instance, MYC has been shown to repress p53 by targeting p53-MDM2-ARF (Kung and Weber, 2022). MYC activates the expression of SENEBLOC, a lncRNA that acts as a scaffold to facilitate the binding of MDM2 with p53, leading to the downregulation of p53 (Xu et al., 2020). Furthermore, MYC also drives the expression of MILIP, another lncRNA that represses p53 by promoting p53 turnover by reducing p53 sumoylation (Feng et al., 2020). Therefore, it is essential to consider the indirect effects mediated by MYC-induced transcription amplification when studying the repression of MYC target genes (Lin et al., 2012; Nie et al., 2012).

## Role of MYC in embryogenesis, cell cycle, proliferation, and apoptosis

As discussed above, MYC is an integral part of transcription progression, acting as a global amplifier, it is indispensable for both embryonic development and the maintenance of self-renewing tissues in adults (Yoshida, 2018). MYC proteins exert crucial functions mostly during embryogenesis and in tissue regenerative programs in adults (Dang, 2013; Asami et al., 2022; Asami et al., 2023). MYC was absolutely required for the immediate embryonic gene activation (iEGA). Inhibiting MYC during iEGA resulted in acute developmental arrest and caused a failure in activating approximately 95% of the upregulated genes. Further, it also changes the morphology of the embryo, and hindered the process of cytokinesis (Asami et al., 2023). In the absence of MYC, the failure of activation of 95% upregulated genes supports the notion that MYC acts as a global amplifier in developmental contexts (Lin et al., 2012; Nie et al., 2012; Nie et al., 2020). Studies have shown that knockouts of either MYC or MYCN do not survive embryonic development, whereas mice lacking MYCL are fertile and appear to develop normally (Charron et al., 1992; Stanton et al., 1992; Davis et al., 1993; Hatton et al., 1996). Mouse embryos lacking MYC experience prenatal mortality at E10.5 due to placental defects (Davis et al., 1993). However, when MYC was deleted in epiblast, the embryos demonstrate normal growth and survive until E11.5, and later develop hematopoiesis and die (Dubois et al., 2008). MYC is typically expressed at low levels, and elevated expression is almost always transient in normal cells (Levens, 2013). Deletion of certain enhancer regions that regulate MYC expression (discussed in regulation section) have examined a role for MYC in embryogenesis (Dave et al., 2017). Upon deletion of an enhancer region, MYC levels reduce by approximately 50%, but are still sufficient for normal development and tissue growth suggesting that the deleted regions were dispensable for MYC function in the placenta development and during early hematopoiesis. These mice were resistant to tumor formation suggesting that tumors demand elevated MYC levels (Dave et al., 2017). Moreover, the enhancer region known as BENC that regulates MYC abundance, plays a crucial role in precisely regulating hematopoiesis (Bahr et al., 2018). These results support that physiological levels of MYC are a crucial factor in regulating embryogenesis.

MYC helps to regulate the cell-cycle and determine the rate of proliferation. Low MYC promotes growth of quiescence cells and controls cell cycle entrance and exit. The G1 and G2 phases of the cell cycle are lengthened in MYC-deficient rat fibroblasts compared to wild-type cells (Mateyak et al., 1997). MYC depletion using antisense oligodeoxynucleotides in human lymphoid and myeloid cells hinders entry into S-phase (Heikkila et al., 1987; Wickstrom et al., 1988). Depletion of MYC using short-hairpin RNA (sh-RNA) led to cell-cycle arrest in the G0/G1 phase in all non-transformed cells, whereas barring few, most transformed cells showed arrest in either the S phase or the G2/M phase (Wang et al., 2008). MYC regulates the expression of genes involved in cell-cycle control by activating the expression of positive regulators of cell-cycle such as Cyclin D, CDK (CDK1, 2, 4, 6), Cyclin E, Cyclin B. MYC also activates E2F target genes (Bretones et al., 2015; García-Gutiérrez et al., 2019). In addition, MYC also exerts its effect by inhibiting the negative regulators of the cell cycle, such as p15, p21, and p27 (Bretones et al., 2015; García-Gutiérrez et al., 2019). MYC represses p15 by forming a repressor complex with SP1 and SMAD in the presence of TGF-β (Seoane et al., 2001; Feng et al., 2016). Another prominent target of MYC is p21. The Interaction between MYC and MIZ-1 leads to the displacement of the transcriptional coactivators, resulting in the inhibition of MIZ-1 target genes like p21 (Wiese et al., 2013; Walz et al., 2014). Further, MYC induces the bHLH-LZ transcription factor AP4 which binds to p21 promoter and facilitates the transcriptional repression of p21 (Jung et al., 2008). It also represses p21 by activating the expression of microRNA miR-17-92 (Wong et al., 2010). MYC represses p27 at both the transcriptional and post-transcriptional levels reviewed in Ahmadi et al., 2021. MYC induces the expression of D-type cyclin, CDK4, CDK6, and components of the SCF^SKP2^ ubiquitin ligase complex, which direct the phosphorylation, degradation, and proteasome-mediated turnover of p27 (Montagnoli et al., 1999; Keller et al., 2007; Bretones et al., 2011). It should be noted that in no case has MYC been shown to directly block the expression of a cell-cycle repressor other than in specific combination with other transcription factors. Mostly simply, MYC regulates the cell-cycle, growth, and proliferation as a general amplifier of preexisting transcriptional programs inducing the expression of required genes in a timely manner.

Beyond its role in cell cycle growth and proliferation, MYC also plays a part in apoptosis. The involvement of MYC in apoptosis first became apparent in a study where elevated MYC led to apoptosis of growth factor-deprived fibroblasts (Evan et al., 1992). MYC controls apoptosis by modulating the balance between pro-survival and pro-apoptotic signals in the BCL pathway (McMahon, 2014). While modest increases in MYC levels led to increased cellular proliferation, higher MYC levels provoked apoptosis (Murphy et al., 2008). Even in normal physiological contexts, endogenous MYC was found to be an essential factor for apoptosis of self-reactive lymphocytes (Shi et al., 1992). Further, it has been shown that endogenous MYC is required for p53-mediated apoptosis in intestinal epithelial cells of mice (Phesse et al., 2014). These studies highlighted that endogenous levels of MYC maybe sufficient to induce apoptosis and based on cellular demands, nutrient levels, growth factors, *etc.* MYC can activate both p53-dependent and -independent apoptosis (Topham et al., 2015). In situations where pro-apoptotic genes are silent, the transcription of those pro-apoptotic genes must be primed before MYC further amplify their expression leading to apoptosis (Lin et al., 2012; Nie et al., 2012; 2020).

## MYC in transcription and replication

MYC binds the genes transcribed by all three RNAPs- I, II, and III although with relatively lower binding to rRNA promoters (Gomez-Roman et al., 2003; Grandori et al., 2005; Oskarsson and Trumpp, 2005). MYC regulates the expression of non-coding transcripts by RNAP I and III, and most prominently mRNA expression by RNAP II (Baluapuri et al., 2019). The chromatin landscape of MYC binding sites indicates that it tends to bind primarily to active promoters or promoters linked to a preoccupied basal transcription apparatus. MYC exhibits a strong association with factors regulating RNAP II activity, including both promoter recruitment and activation of the polymerase. It directly binds to the TATA-binding protein (TBP), an essential component of the TFIID complex responsible for promoter recognition and pre-initiation complex assembly at the transcriptional start site (Wei et al., 2019). This interaction suggests a potential mechanism for TBP recruitment to MYC targets lacking a TATA box.

The rate-limiting step of transcriptional initiation, which involves the phosphorylation of Ser5 in the RNAP II C-terminal domain, is regulated by the recruitment of SPT5/SPT6, the two components of DSIF, through the influence of MYC. MYC interacts with SPT5, facilitating its recruitment to promoters and subsequent CDK7-dependent transfer to the RNAP II prior to transcription elongation. This process enables SPT5-loaded RNAP II to efficiently generate full-length transcripts through fast, continuous, and directed transcription (Baluapuri et al., 2019). When MYC is low (quiescent cells), the recruitment of SPT5 at RNAP II is insufficient, leading to a loss of directionality and processivity in RNAP II, which results in elevated production of antisense and abortive transcripts. However, it remains to elucidate the biological consequence of these antisense and abortive transcripts.

Further, MYC facilitates the formation of the P-TEFb complex and phosphorylation of Ser2 on the RNAP II CTD, to promote transcription elongation (Yu et al., 2018; Guan et al., 2023). MYC-dependent transcription activation also requires ubiquitination of MYC. It was shown that ubiquitylation of MYC is required to transfer of the PAF1c from the MYC to transcription elongation complex (otherwise repressive complex) onto RNAP II (Jaenicke et al., 2016). However, it remained unclear whether MYC ubiquitination alone was sufficient for the transfer or if it also required the involvement of P-TEFb. Excitingly, recently it has been shown that MYC recruits the PAF1c complex, and in conjunction with HUWE1-mediated ubiquitylation of MYC at the promoter, facilitates the transfer of PAF1c from MYC to RNAP II (Figure 2). This event triggers promoter escape and enables continuous elongation, which occurs downstream of the P-TEFb-dependent release of RNAP II from NELF inhibition (Endres et al., 2021). The elimination of MYC from genes is facilitated by E3-directed poly-ubiquitin pathways, which could be closely linked to its role in regulating transcription activation and amplification. A recent study proposes that increased MYC leads to its invasion of super-enhancers (See et al., 2022). MYC utilizes various members of the KLF/SP transcription factor family, such as MAZ, ZBTB17, and EGR2 at super-enhancers. MYC interaction with super-enhancers increased the chromatin contact frequency across TADs boundaries. Further, increased MYC levels strengthen chromatin interactions between MYC binding sites at promoters and enhancers.

With MYC-driven transcription amplification, torsional stress builds up. If torsional stress is not resolved, it would quickly hinder the movement of RNAP II and stop bursts of transcription, as in bacteria (Chong et al., 2014). To maintain a high level of transcription, it is crucial to promptly reduce torsional stress (Jha et al., 2022). If MYC-driven transcription were accompanied by an increase in torsional resistance, the speed of transcription would slow down or even stop, counteracting any efforts made by MYC to boost transcription output. MYC topoisome, a recently discovered complex is a crucial regulator for the maintenance of transcription-induced torsional stress in such situations. MYC interacts with TOP1 and TOP2A and forms the MYC topoisome complex (Figure 2) in which the catalytic activities of both TOP1 and TOP2A are increased to facilitate transcription (Das et al., 2022). Notably MYCN forms a distinct topoisome incorporating TOP1 and TOP2B.

Apart from torsional stress, MYC-driven transcription amplification can also increase the chance of transcription-replication conflict. A recent finding shows that MYC forms multimers, which suppress transcription-replication conflicts (T-R conflicts) and DNA damage (Solvie et al., 2022). Through super-resolution microscopic analysis of the MYC distribution in cells revealed foci of MYC multimers. These multimers consisted of a dense MYC shell surrounding a weakly stained core. Regulators of proteasome inhibition, ubiquitylation, splicing, and transcription elongation were found to influence the formation of MYC multimers. MYC multimers drive away SPT5 from RNAP II, attenuating MYC-dependent transcription. FANCD2 and BRCA1, associated with stalled replication forks in multimers were localized near replication forks to prevent T-R conflicts. Further, HUWE1-mediated MYC polyubiquitylation drove multimerization, suppressing antisense transcription, replication-fork degradation, and double-strand DNA break formation (Solvie et al., 2022).

MYC has been shown to regulate rDNA transcription. MYC interacts with components of the SL1 complex, enhancing the association of TBP and TAF complex with the promoter and recruiting HATs to facilitate RNAP I recruitment and transcription at rDNA promoters. Consequently, the upregulation of UBF expression mediated by MYC positively influences the transcriptional activity of RNAP I, ultimately resulting in enhanced rRNA synthesis (Grandori et al., 2005; Grewal et al., 2005; Oskarsson and Trumpp, 2005). Sumoylation of MYC has been shown to regulate the MYC-mediated transcription by RNAP I as well. Sumoylation marks MYC for degradation through the proteasome pathway (Peng et al., 2019), this degradation mechanism counteracts the potential transcriptional MYC-mediated activation of RNAP I. It has been speculated MYC functions as a coordinator during differentiation, aligning the pool of active rRNA genes with the levels of RNAP I factors to tightly regulate rDNA transcription. This orchestration of gene expression ensures the proper synthesis of ribosomes to meet the changing needs of the cell throughout its differentiation process (Poortinga et al., 2011).

MYC proteins are intrinsically disordered proteins (IDPs). They tend to interact with different proteins simultaneously and has been speculated that MYC forms liquid-liquid phase separation when present at high concentration (Ann Boija et al., 2018). It has been reported that MYCN can form condensates that may be transcriptionally active, and the IDR and DNA binding domain of MYCN seem to be critical for such condensates in neuroblastoma cells (Yang et al., 2022). However, the impact of MYCN condensates on the transcriptome appears to be limited, as fewer than 6% of genes were altered among the numerous transcripts dependent on MYCN. Overall, further investigation is warranted to determine mechanisms involved for MYC condensate formation and explore its effect on gene regulation, and involvement in disease conditions if any.

## Regulation of MYC

**FIGURE 3:**
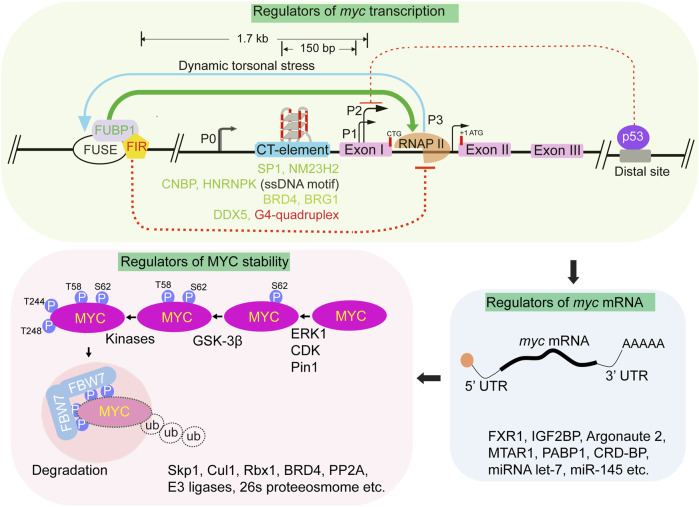
Regulation of MYC. Schematic depicting the various layers in regulation of MYC cellular levels. At the transcription level, multiple promoters (P0, P1, P2 and P3, not drawn on scale) participate in *myc* transcription. Primary *myc* transcription predominantly initiates from two major promoters, P1 and P2, contributing to roughly 10%–25% and 75%–90% of *myc* mRNA, respectively. The MYC promoter is regulated by two noncanonical cis-regulatory elements: FUSE and the CT element, induced by negative supercoiling generated during transcription activation. The FUSE element is located 1.7 kb upstream of P2, while the CT element is located between −100- and −150 bp upstream of P1. The FUSE element, which is AT-rich, melts in response to torsional stress caused by transcription activation. FUSE melting facilitates sequence-specific FUBP1 binding. Dynamic changes in DNA supercoiling regulates FUBP1 and FIR binding to the FUSE element with FUBP1 positively (Green arrow) and FIR negatively (Red dotted line) influencing *myc* transcription. The CT element which is GC-rich, facilitates the formation of alternate DNA structures. Numerous transcription factors like SP1, NM23H2, CNBP, HNRNPK, and DDX5 bind to CT element and regulate MYC transcription. Non-B DNA structures, such as G-quadruplex can form at CT elements and negatively regulate *myc* transcription. The binding of BRD4 throughout the promoter regions positively regulates *myc* transcription*.* Factors like Brg1 and BRD4 regulate myc transcription by influencing the interaction between enhancer and promoter regions*.* The binding of p53 to a distal region of MYC repress *myc* transcription. Multiple factors including RNA, RNA binding proteins and long noncoding RNAs (as indicated), regulate post-transcriptional regulation of *myc* mRNA. MYC levels are further regulated by various factors (as listed) and post-transcriptional modifications. MYC phosphorylation by known or unknown kinases at specific site sets the stage for MYC degradation. Phosphorylation of indicated sites recruit FBW7 dimer and forms the SCF complex consisting of Skp1, Cul1 and Rbx1 proteins followed by polyubiquitination of MYC and degradation by the 26s proteosome.

Due to its relatively unstable mRNA and protein, MYC acts as a highly efficient regulator of rapid cellular responses. MYC has one of the shortest mRNA half-lives, approximately 10–20 min (Dani et al., 1984) and protein half-lives, approximately 20 min (Hann and Eisenman, 1984), there are various mechanisms that have been shown to regulate MYC level. The regulation of MYC expression involves signalling pathways that operate at the transcriptional, post-transcriptional, and protein levels by a range of upstream and downstream mechanisms (Figure 3) (Levens, 2013). The MYC gene is transcribed from multiple promoters (P0, P1, P2, and P3), and uses different initiation sites, alternative polyadenylation sites, and the production of antisense transcripts (Nepveu et al., 1987; Chung and Levens, 2005). The mRNA transcribed by the P1 promoter represents 10%–25% of all *myc* mRNA transcripts, while the P2 promoter accounts for 75%–90% of the transcripts (Figure 3). Promoter P2 requires the presence of specific elements for initiating *c-myc* gene transcription (Hay et al., 1987; Moberg et al., 1991; Liu and Levens, 2006). The regulation of the *c-myc* locus involves DNA-level modulation through alternate non-B DNA structures (Levens, 2010). In the typical cellular environment, DNA primarily adopts the B-form, which is a classical right-handed double helix. However, a variety of non-B DNA structures have been reported both *in vitro* and *in vivo* with evident regulatory potential (Zaytseva and Quinn, 2018). One such example includes the Far Up Stream Element (FUSE) of the human MYC gene, the FUSE in the MYC promoter responds to negative supercoiling forces during transcription (Figure 3). Dynamic changes in DNA conformation are coupled with promoter output and are recognized by transcriptional factors, FIR (FUBP interacting repressor) and FUSE-binding protein (FUBP1). Transcription-generated DNA supercoiling induced melting of the FUSE region, recruits FUBP1 and the FIR to regulate the advancement of the transcription machinery through TFIIH activation. As transcription levels increase, FUBP1 facilitates progression through pausing, while further melting of FUSE recruits FIR, ultimately restoring MYC expression to basal levels (Figure 3) (Liu et al., 2006; Kouzine et al., 2008). Apart from FUBP1-FIR mediated regulation, the negative supercoiling generated during transcription can induce dynamic changes and facilitate the formation of G-quadruplexes (G4s) in the CT element region of the MYC promoter. G4 structure forms in the MYC promoter region and may impede MYC transcription by obstructing the binding of transcriptional factors, including double-stranded factor SP1, single-stranded factors CNBP, and hnRNPK (Figure 3) (Michelotti et al., 1996a). A study shows that DDX5, a potent resolvase of DNA and RNA G4s structures, unfold G4 at the MYC promoter and hence increases the MYC transcription in the cell (Wu et al., 2019, PNAS). However, the role of G4 is uncertain as it has also been claimed to activate MYC transcription (Hänsel-Hertsch et al., 2016).

These multiple transcription factors and chromatin regulators have been shown to regulate MYC expression in response to various signals. Fine-tuned control of MYC expression is dependent on sets of enhancers positioned both upstream and downstream of the gene. The *c-myc* gene is positioned within approximately a 3 Mb region that lacks other protein-coding genes and corresponds to a single topologically associating domain (MYC-TAD). The MYC-TAD harbors a multitude of super-enhancer regions that intricately regulate the expression of the MYC (Sur et al., 2012; Kieffer-Kwon et al., 2013; Uslu et al., 2014; Yashiro-Ohtani et al., 2014). These enhancers include tissue-specific enhancers that respond to diverse stimuli, along with super-enhancers (Lancho and Herranz, 2018). Removal of an enhancer present over half a megabase of DNA upstream of the *c-myc* gene (one of several different regions that have been called super-enhancers) led to a ∼50% reduced MYC level (Dave et al., 2017). A MYC super-enhancer located approximately 1.7 Mb downstream of the transcription start site plays a critical role in tightly controlling MYC expression and promoting increased chromatin accessibility (Shi et al., 2013; Mifsud et al., 2015; Bahr et al., 2018; Jia et al., 2019). The enhancer region (termed BENC) is required for MYC expression, it consists of enhancer modules that are specific to cell lineages. When these modules are deleted, it results in the downregulation of MYC expression in a cell-type-specific manner precisely correlating with gene expression (Bahr et al., 2018).

It has been shown that p53 (tumor-suppressor) regulates the expression of MYC by binding to ∼50 kb downstream of the *c-myc* locus. It has been suggested that p53 binding at this site represses MYC through the involvement of a MYC enhancer (Figure 3) (Porter et al., 2017). A recent study shows that ATM represses MYC expression by promoting transcriptional-induced DNA repair at the MYC enhancer region (Najnin et al., 2023). Further, MYC regulation through enhancers appears to be a complex process and involves multiple regulatory elements, chromatin remodeling factors, RNA, and RNA binding proteins (Figure 3). For instance, FXR1 (RNA binding protein) binds to the AU rich elements (ARE) within the 3′ UTR of *myc* mRNA and improves its stability (George et al., 2021). The RGG domain of FXR1 interacts with eIF4A1 and eIF4E and facilitates recruitment of the eIF4F complex to translation initiation sites for cMYC translation ultimately increasing the total level of MYC in the cells (George et al., 2021). Another RNA binding protein, IGF2BP, can recognize and bind m^6^A modified-*myc* mRNA to regulate its translation (Huang et al., 2018). MTAR1, a long noncoding RNA has been shown to facilitate IGF2BP-meditated MYC translation (Gao et al., 2022). Further, a point mutation within the intron of long noncoding RNA CCDC26 plays a role in regulating MYC expression (Yanchus et al., 2022). A risk SNP allele in a brain specific enhancer almost 2 megabase 3’ of MYC, rs55705857(G), disrupts OCT2/4 binding that otherwise decreases interactions with the MYC promoter. Consequently, this SNP positively influences the regulation of MYC expression (Yanchus et al., 2022). The RNA-binding protein Argonaute 2, known for its involvement in the RNA-induced silencing complex, has been found to directly bind and stabilize *myc* mRNA (Zhang et al., 2020). RNA-binding protein hnRNPK, also controls MYC expression by directly binding to the CT-element and interacting with the transcription machinery (Figure 3) (Michelotti et al., 1996a; Michelotti et al., 1996b). Further, a recent study shows that RNA molecules originating from both MYC enhancers and promoter interact with the hnRNPK. Through its oligomerization, hnRNPK brings the MYC enhancer and promoter in proximity, thereby facilitating the elevated level of MYC (Cai et al., 2020).

The MYC amplifier role is dependent on cellular MYC levels. Slight increases in MYC levels have been shown to release cells from cell-cycle arrest, promote proliferation or trigger apoptosis. MYC levels have been observed to show an inverse correlation with cell cycle length and a direct correlation with organism size within a species (Murphy et al., 2008; Shachaf et al., 2008). Studies utilizing genetic approaches in *Drosophila* have demonstrated using developmental compartments containing a mixture of normal cells with cells expressing either double or half the normal levels of MYC, elimination of the lower MYC cells. High-MYC cells then expand, refill the compartment, and undergo normal development (De La Cova et al., 2004; Moreno and Basler, 2004; Johnston, 2014; Topham et al., 2015). The elimination of low-MYC cells in favor of high-MYC cells is termed supercompetition and underscores the critical importance of MYC levels in determining cellular fate. A recent study using exogenously expressed MYC tagged with the fluorescent protein mNeonGreen (mNG) showed that MYC expression is pulsatile, heterogeneous, and dependent on MAPK and Wnt signaling pathways (Liu et al., 2023). The heterogeneous expression of MYC leads to variable gene transcription and variable cell-cycle progression rates. Cells with high MYC, progress to S-phase rapidly and cells with low MYC have increased G0/G1 length, and so transcriptome diversity arises in the previously homogenous population. MYC, which regulates G0/G1 length and other processes, influences sensitivity to chemotherapy drugs. Reduction in MYC protein levels during doxorubicin (a chemotherapeutic agent that target topoisomerase II) treatment increased the number of surviving cells. Cells with transiently low MYC levels at the time of drug treatment were more likely to survive and proliferate. Even among cells that remained in G0/G1 throughout drug treatment, those with lower MYC immediately after treatment had higher chances of survival and proliferation. This indicates that low MYC levels limit DNA damage during gene expression. It is suggested that increasing heterogeneity of MYC may be advantageous for cancers (Liu et al., 2023). However, whether normal cells also possess heterogeneity in MYC expression and the consequences of that heterogeneity in normal physiological conditions is a matter of investigation.

The level of MYC in cells is also controlled by post-translational mechanisms such as MYC phosphorylation which plays a crucial role in controlling its turnover and degradation as recently reviewed (Sun et al., 2021). The highly conserved serine and threonine residues in MBI T58, S62, S64 and S67 undergo phosphorylation (Welcker et al., 2004). ERK kinase phosphorylates S62 within the MYC transactivation domain and enhances the stability of MYC. In contrast, GSK3β or BRD4 kinases phosphorylates MYC at threonine 58 (T58) promotes degradation of MYC (Sears et al., 2000). The dually phosphorylated form of MYC, with both S62 and T58 phosphorylation, is recognized by the phosphatase PP2A which removes S62 phosphorylation, and this event primes the recruitment of an E3 ubiquitin ligase called FBW7 (F-box/WD repeat-containing protein 7). FBW7 recognizes phosphorylated MYC and facilitates its ubiquitination, marking it for proteasomal degradation (Sears et al., 2000; Yeh et al., 2004; Arnold and Sears, 2006). However, this long-standing model for MYC degradation has been countered by a recent finding, where authors show phosphorylation of S62 does not stabilize MYC by preventing FBW7 from binding to it (Welcker et al., 2022). Instead, it enhances the interaction between MYC and FBW7, leading to degradation of MYC. Furthermore, a previously unknown dephosphorylated degron at residues T244/T248 was identified that also promotes MYC binding to FBW7. This additional degron acts alongside the T58/S62 phosphorylation to regulate MYC protein levels (Figure 3) (Welcker et al., 2022). This finding supports that stabilizing effects of pS62 may be independent of FBW7 binding (Vaseva et al., 2018) and highlight the complexity of MYC regulation and suggest that S62 phosphorylation has multiple roles beyond FBW7 binding, influencing MYC stability and function. BRD4 also regulates MYC levels by both degradation and transcriptional activation of MYC (Figure 3) (Devaiah et al., 2020). Given the significant impact of MYC levels on cellular behavior, it is crucial to understand the underlying mechanistic processes and how MYC levels are regulated. These questions remain a subject of intense investigation.

## Approaches to tackle MYC

MYC is elevated in most cancers and several other pathological conditions, and so has been proposed as a drug target for decades. However, due to MYC being a general transcription amplifier in both normal and cancerous cells, directly targeting it has proven challenging. Further, MYC has “undruggable characteristics” such as the absence of an enzymatic pocket for small molecules to bind, and its predominantly nuclear localization hinders antibody access. Recent promising studies highlighted that partial depletion or inhibition of MYC may be sufficient for treatment of MYC-dependent cancers and other diseases (Hofmann et al., 2015; Wang et al., 2021). The current approaches to tackle MYC-dependent pathogenesis fall into various categories such as downregulating MYC at the transcriptional or post-transcriptional levels, and hindrance of MYC-MAX interaction.

There are many approaches to inhibit MYC at the transcription level. Inhibitors like QN-1, a difluoro-substituted quinoxaline, APTO-253, a selective p21 inducer, and CX-3543, quarfloxin, are G-quadruplex stabilizers. These inhibitors specifically stabilize the G-quadruplex at the MYC promoter and in turn repress MYC expression (Cercek et al., 2015; Local et al., 2018; Hu et al., 2019; Paul et al., 2020). Although, APTO-253 was in clinical trial for acute myeloid leukemia and high-risk myelodysplastic syndrome, it was terminated due to it lack efficacy in a phase 1. MYC expression can be targeted by inhibiting factors that activate MYC transcription, such as DDX5, BRD4, and SWI/SNF. DDX5 has been shown to activate MYC transcription by resolving G-quadruplex formation at the MYC promoter (Wu et al., 2019), thus inhibiting DDX5 might have a favorable effect on MYC-dependent diseases. Inhibitors like AZD5153, GSK525762, JQ1, and dBET1 repress MYC expression by targeting BRD4 (Wang et al., 2021). Brg1, an ATPase subunit of SWI/SNF positively regulates MYC expression by binding to an enhancer region of MYC (Shi et al., 2013). Knockdown of Brg1 or its inhibition with an ATPase inhibitor BRM014 disrupts the BENC enhancer cluster and represses MYC expression (Shi et al., 2013; Bahr et al., 2018; Rago et al., 2022; Chambers et al., 2023). These results promise continued development of SWI/SNF inhibitors in the treatment of MYC-dependent cancers and other diseases.

Another approach is to target MYC protein by direct binding-ligands. Despite, MYC lacking a precise ligand binding pocket, a recent study has emphasized the effectiveness of covalent ligand compounds that target IDR regions of MYC. For instance, EN4 is a compound that primarily interacts with cysteine (C171) within the IDR region of MYC, thereby reducing the thermal stability of MYC-MAX dimerization and subsequently its function (Boike et al., 2021). MYC functions have been indirectly challenged by targeting MYC- MAX heterodimerization. KI-MS2-008 is a drug that stabilizes the MAX homodimer to prevent MYC-MAX interaction (Struntz et al., 2019). Similarly, Omomyc (bHLH-zip domain of MYC with 4 mutations) binds to MYC bHLH-zip domain and prevents its interactions. MYCi975 is a small molecule inhibitor, which binds MYC directly to disrupt MYC-MAX interaction and increases the proteasomal degradation of MYC, and thus leads to decreased tumor growth (Han et al., 2019; Truica et al., 2021). Further, MYCi975 alters the binding of MYC as well as MYC network proteins like MAX to chromatin (Holmes et al., 2022). While the prospect of disrupting the MYC-MAX heterodimer, either by dismantling it or occupying the binding interface between the two proteins, holds promise as an alternative strategy for targeting MYC, it is important to note that the complete inhibition of MYC function by dimerization inhibition could have adverse effects on normal cells. Therefore, another approach could be the targeting of the MYC-partners that mediate its function. Recently, a specific TFIIS N-terminal domains (TNDs) and unstructured TND-interacting motifs (TIMs) binary interaction module has been established, and this module is conserved for many transcription factors including PP1-PNUTS5 (Cermakova et al., 2021; Cermakova et al., 2023). MYC protein is stabilized by the PP1 phosphatase and its regulatory subunit PNUTS. It has been shown that PNUTS interacts with MB0, and controls MYC phosphorylation, chromatin eviction, and MYC degradation. Disrupting the PNUTS-MYC interaction would enhance MYC degradation (Wei et al., 2022). This could be a new avenue to explore to limit MYC function and MYC-dependent pathological activity.

## Future perspective

MYC protein is a crucial transcription regulator that plays a central role in regulating gene expression in different cellular situations. Its capacity to amplify transcriptional responses contributes to the precise control of cellular processes and the maintenance of a balanced state within cells. It is not known whether MYC exerts its pathological action from an augmentation of its normal transcription amplifier role or whether MYC neopathologic functions are elicited at supraphysiological levels. It is important to understand mechanistically how MYC regulates different kinetic steps of transcription by all three RNAPs. A deeper understanding of the mechanisms through which MYC amplifies transcription, and the factors that influence this process in physiological and pathological conditions will enhance our knowledge of gene regulation and offer valuable insights for developing targeted therapeutic approaches for MYC-related disorders.
